# Compliance with NICE, BAUS, RCR, and RCEM Guidelines for Acute Renal Colic: A Two-Cycle Quality Improvement Study at Lister Hospital Emergency Department, United Kingdom

**DOI:** 10.7759/cureus.93387

**Published:** 2025-09-28

**Authors:** Aabid Nehvi, Audrey Ofedie, Syed Maaz Salahuddin, Fahad Akhtar, Binay Shrestha, Shakeel Faruqui

**Affiliations:** 1 Emergency Medicine, East and North Hertfordshire Teaching NHS Trust, Stevenage, GBR; 2 Radiology, East and North Hertfordshire Teaching NHS Trust, Stevenage, GBR

**Keywords:** acute renal colic, baus guidelines, ct-kub, emergency department efficiency, nice guidelines, quality improvement, rcem four-hour standard, rcr irefer, stone score

## Abstract

Background: Acute renal colic is a common emergency department (ED) presentation requiring rapid diagnosis and management. National bodies emphasize timely imaging, appropriate biochemical testing, and ED throughput standards.

Objective: To evaluate and improve compliance with the National Institute for Health and Care Excellence (NICE), British Association of Urological Surgeons (BAUS), Royal College of Radiologists (RCR), and Royal College of Emergency Medicine (RCEM) standards in the ED management of acute renal colic at a United Kingdom (UK) district general hospital, with the following prespecified targets: RCEM four-hour compliance ≥90% (local “green” standard); median time from ED arrival to CT-KUB (CT scan of the kidneys, ureters, and bladder) request ≤90 minutes and CT report ≤90 minutes; >90% of scans within BAUS/NICE imaging timeframes (≤14 h/≤24 h); STONE (sex, timing, origin, nausea, erythrocytes) score documentation ≥75% and serum calcium testing ≥65% (with serum urate ≥20%); and ≥30% reduction in repeat CT within three months.

Methods: A retrospective two-cycle quality improvement project (QIP) was conducted. Cycle 1 (December 2023-March 2024) included 114 consecutive adults with suspected renal colic; Cycle 2 (March-August 2024, n = 114) assessed post-intervention impact. Metrics included urine dip turnaround, CT-KUB request and reporting times, RCEM four-hour compliance, serum testing, STONE score use, repeat CT rates, diagnostic yield, and length of stay. Interventions comprised a “Straight to CT” pathway, Integrated Clinical Environment (ICE)/iRefer STONE prompts, staff education, and workflow optimization. Categorical variables were analyzed using chi-square or Fisher’s exact tests; continuous outcomes were reported as median (interquartile range (IQR)) and compared using Mann-Whitney U tests.

Results: Cycle 1 showed prolonged urine dip turnaround (median: 144 minutes), CT request (166 minutes), and report (124 minutes), with RCEM four-hour compliance as 16.7% (19/114). Cycle 2 improved significantly: urine dip 68 minutes (U=2102; p<0.001), CT request 75 minutes (U=1850; p<0.001), CT report 70 minutes (U=1924; p<0.001), RCEM compliance 57% (χ²=39.9; p<0.001; odds ratio (OR) 6.63 95% confidence interval (CI): 3.58-12.29). Serum calcium testing improved (χ²=5.27; p=0.022), urate testing (Fisher’s exact p<0.001), STONE score use (χ²=127.0; p<0.001), and repeat CT <3 months halved (RR: 0.50, 95% CI: 0.25-0.98; p=0.038; CI width noted for cautious interpretation). Diagnostic yield showed an upward trend (68.4% vs 57.9%; p=0.090) and alternative diagnoses a decreasing trend (7.0% vs 11.4%; p=0.252). Median ED stay reduced from 7.5 to 3.9 hours (U=1765; p<0.001).

Conclusion: This QIP demonstrated that targeted interventions significantly enhanced adherence to NICE, BAUS, RCR, and RCEM guidelines, reduced diagnostic delays, and improved emergency department efficiency in managing acute renal colic. Scalable strategies, including STONE score integration and streamlined imaging, expedited workflows, increased RCEM four-hour compliance, and boosted metabolic testing rates while reducing repeat CT scans. Non-significant trends in diagnostic yield and alternative diagnoses suggest improved clinical precision without compromising safety, offering a replicable model for optimizing renal colic care within existing resources, pending further multicenter validation.

## Introduction

Acute renal colic is a common emergency department presentation, with a lifetime prevalence of up to 15% [1]. Prompt diagnosis and management are critical to alleviate pain and prevent complications such as obstruction or infection. National guidelines provide clear standards for acute renal colic management. The National Institute for Health and Care Excellence (NICE) guideline NG118 (January 2019; reviewed February 2021) recommends low-dose non-contrast CT-KUB (CT scan of the kidneys, ureters, and bladder) as the first-line imaging modality, with NICE Quality Standard QS195 (August 2020; updated February 2024) mandating imaging within 24 hours of presentation and nonsteroidal anti-inflammatory drugs (NSAIDs) as first-line analgesia, alongside serum calcium testing for metabolic assessment [2,3]. The British Association of Urological Surgeons (BAUS, 2018) advises CT-KUB within 14 hours, a biochemical profile including serum calcium and urate, and avoiding repeat CT within three months for known stone formers, preferring ultrasound or plain X-ray to minimize radiation exposure [4]. The Royal College of Radiologists (RCR) iRefer guidelines establish CT-KUB as the gold standard. Published UK studies report diagnostic yields of 44-64% for calculi and 6-18% for alternative diagnoses [5]. The Royal College of Emergency Medicine (RCEM) sets a target that 95% or more of ED patients be admitted, transferred, or discharged within four hours [6]. The STONE (sex, timing, origin, nausea, erythrocytes) score, a validated clinical prediction rule, aids risk stratification and can reduce unnecessary radiation exposure [7]. Despite clear guidance, audits and service evaluations commonly show delays in imaging, incomplete metabolic testing, and challenges in meeting the RCEM four-hour standard [8-12]. Evidence suggests that structured pathways, such as “Straight-to-CT” for appropriately selected patients, electronic decision support (e.g., Integrated Clinical Environment (ICE)/iRefer prompts), and targeted staff education can improve diagnostic yield, reduce turnaround times, and limit repeat CT within short intervals [9-12,13-15].

A preliminary audit at Lister Hospital, United Kingdom, identified prolonged urine dip and CT-KUB turnaround times, low STONE score documentation, incomplete biochemical testing, and suboptimal RCEM four-hour compliance. This quality improvement project (QIP) therefore aimed to (i) evaluate baseline compliance with NICE, BAUS, RCR, and RCEM standards and (ii) implement and assess a multi-component intervention to improve timeliness, diagnostic efficiency, and ED throughput for suspected renal colic.

## Materials and methods

Design and setting

We conducted a retrospective two-cycle QIP in the ED of Lister Hospital, East and North Hertfordshire National Health Service (NHS) Trust (United Kingdom). The hospital serves a catchment of approximately 600,000 people with more than 100,000 annual ED attendances. A multidisciplinary team - including ED clinicians, radiologists, urologists, and administrative staff - designed and implemented the project using a Plan-Do-Study-Act (PDSA) approach.

Eligibility and cohorts

Inclusion criteria: Adults aged ≥18 years with suspected renal or ureteral colic (flank or loin-to-groin pain with or without hematuria) at the index ED visit. Patient inclusion was consecutive, and eligibility criteria were applied consistently across both cycles.

Exclusion criteria: Pregnant patients or those with incomplete electronic health records (EHR).

Cohorts: Consecutive eligible patients were included: Cycle 1 (December 2023-March 2024; n=114) and Cycle 2 (March-August 2024; n=114). No formal power calculation was performed; the sample size was pragmatic, based on expected case volume.

Case ascertainment and data sources

Patients were identified through systematic queries of the EHR (Cerner Millennium), radiology information system (RIS)/Picture Archiving and Communication System (PACS), ED attendance logs, and laboratory records using predefined keywords (e.g., “renal colic”, “ureteral stone”, “flank pain”). Cross-checking confirmed suspected colic cases with clinical and/or radiological findings.

Data abstraction and quality assurance

Two independent abstractors used a standardized codebook with predefined variable definitions. Disagreements were resolved by consensus with a third reviewer (ED consultant). Missing data (e.g., no recorded CT-KUB) were classified as “not completed.” Data were de-identified and stored on secure Trust servers in accordance with information-governance policies.

Outcomes and operational definitions

Primary outcomes (process measures): Timeliness of CT-KUB - time from ED arrival to CT-KUB request and to availability of the CT report; and RCEM four-hour compliance - proportion of patients discharged or admitted within four hours.

Secondary outcomes: Serum calcium and urate testing; STONE score documentation; repeat CT-KUB within three months; diagnostic yield (stone present on CT); alternative diagnoses; and ED length of stay.

Audit standard

A local compliance threshold of ≥90% was pre-specified as the “green” performance target for the primary standards - imaging timeliness (NICE NG118/QS195; BAUS) and the RCEM four-hour metric - benchmarked against national expectations (RCEM 95%). This target reflects a pragmatic high-reliability aim that allows for unavoidable exceptions (e.g., scanner downtime, patient refusal, inter-hospital transfer, pregnancy requiring alternative imaging), with an intention to progress toward ≥95% as process capability matures. These thresholds were specified a priori as service performance aims and were not used as statistical decision cut-offs.

Target-setting rationale (a priori): Improvement targets were specified before Cycle 1 data extraction, drawing on national standards and operational backtiming of the ED pathway. RCEM’s four-hour standard (95%) informed a local high-reliability “green” threshold of ≥90%, allowing unavoidable exceptions while signaling progression toward ≥95%. To support timely disposition, we prespecified CT-KUB request ≤90 minutes and CT report ≤90 minutes from arrival. For imaging windows, we targeted >90% compliance with BAUS ≤14 hours and NICE QS195 ≤24 hours. Adoption targets reflected expected uptake with mandatory EPR prompts and education: STONE documentation ≥75%; serum calcium ≥65% and serum urate ≥20% as an initial phase-in. A ≥30% reduction in repeat CT within three months was selected a priori based on published QIP effects (≈25-50%). These targets were not derived from baseline data, were not used for sample-size calculation, and were not used as statistical decision thresholds.

Intervention Bundle (Implemented After Cycle 1)

Straight-to-CT pathway: Patients with a STONE score ≥6 or high clinical suspicion were prioritized for immediate CT-KUB without initial ultrasound, unless contraindicated (e.g., pregnancy or low probability). ED registrars were empowered to request scans directly to bypass vetting delays.

ICE/iRefer STONE prompts: Mandatory STONE score fields were integrated into the radiology request workflow in ICE and iRefer, with automated prompts requiring score entry before submission.

Staff education and prompts: Three 20-minute interactive training sessions (weeks one, three, and six) for ED staff covered guideline summaries, STONE calculation, and updated workflows; laminated prompt cards and intranet quick guides reinforced uptake.

Radiology and porter workflow optimization: Dedicated porter slots for urgent CT-KUB transfers and “fast-track” radiology queue markers reduced transport and reporting delays; ED cases received hot reporting during peak hours.

Surgical assessment unit (SAU)/Clinical decision unit (CDU) early transfers: Stable patients were transferred early for urology review, freeing ED capacity and expediting disposition.

These interventions were low cost, leveraging existing systems, and rolled out simultaneously after Cycle 1 completion.

Governance, feedback, and fidelity checks

Weekly run-charts for key processes were reviewed at governance meetings; fortnightly spot-checks (10 random cases) audited STONE documentation, CT-KUB ordering, and discharge documentation with micro-teaching for remediation.

Balancing measures

Potential unintended consequences (e.g., workload shift to radiology, alert fatigue, radiation exposure) were monitored via staff feedback and monthly radiology alert counts; no issues were identified that compromised turnaround times or access to results.

Study of the interventions

A pre-post (two-cycle) design without concurrent controls was used. Cycle 1 defined baseline performance; the intervention bundle was evaluated in Cycle 2. Fidelity metrics and balancing measures were tracked throughout.

Statistical analysis

Proportions were summarized with 95% confidence intervals (CIs) using the Wilson method. Between-cycle differences in categorical outcomes were tested using Pearson’s chi-square (no continuity correction), and Fisher’s exact test was used when any expected cell count was <5. Continuous outcomes were summarized as median (interquartile range, IQR) and compared using Mann-Whitney U tests. Relative risks (RRs) or odds ratios (ORs) with 95% CIs were calculated for key outcomes. A two-sided α = 0.05 was applied. Analyses were performed using Statistical Product and Service Solutions (SPSS, version 27; IBM SPSS Statistics for Windows, Armonk, NY). Normality was not formally assessed and is acknowledged as a limitation; nonparametric tests were therefore used. For outcomes with p≥0.05 but <0.10, we describe findings as non-significant trends and report full effect estimates and 95% CIs.

Ethics and governance

This work was registered as a service evaluation/QIP within the Trust (24/25_Trust_Emergency Med_024253). Formal Research Ethics Committee review was not required; only anonymized routine data were analyzed, with no patient-identifiable results.

The project was presented at the departmental Morbidity and Mortality Meeting and disseminated as a poster at the European Congress of Radiology (ECR 2024).

## Results

Cohorts

Each cycle included 114 consecutive eligible adults with suspected renal colic. In Cycle 1, 82/114 (72%) were discharged from the ED, and 32/114 (28%) were admitted. In Cycle 2, 88/114 (77%) were discharged, and 26/114 (23%) were admitted.

Primary outcomes

Urine dip turnaround decreased from a median of 144 minutes (IQR: 98-192) to 68 minutes (45-92) (U=2102; p<0.001). CT-KUB request time decreased from 166 minutes (120-210) to 75 minutes (50-100) (U=1850; p<0.001). CT-KUB report time decreased from 124 minutes (90-160) to 70 minutes (45-95) (U=1924; p<0.001). RCEM four-hour compliance increased from 19/114 (16.7%; 95% CI: 11-25) to 65/114 (57.0%; 95% CI: 48-66) (χ²=39.9; p<0.001; OR 6.63, 95% CI 3.58-12.29). 

Secondary outcomes

Serum calcium testing increased from 58/114 (50.9%; 95% CI: 42-60) to 75/114 (65.8%; 95% CI: 57-74) (χ²=5.27; p=0.022; RR: 1.29, 95% CI: 1.03-1.62). Serum urate testing increased from 0/114 (0%; 95% CI: 0-3) to 29/114 (25.4%; 95% CI: 18-34) (Fisher’s exact p<0.001). STONE score documentation increased from 6/114 (5.3%; 95% CI: 2-11) to 90/114 (78.9%; 95% CI: 71-85) (χ²=127.0; p<0.001; RR: 15.0, 95% CI: 6.8-32.9). Repeat CT-KUB within three months decreased from 19.3% to 9.6% (χ²=4.31; p=0.038; RR: 0.50, 95% CI: 0.25-0.98); the effect-size CI width warrants cautious interpretation. Diagnostic yield (CT-confirmed stone) increased from 57.9% to 68.4% (χ²=2.87; p=0.090; RR: 1.18, 95% CI: 0.97-1.44), representing an upward trend. ED length of stay decreased from a median of 7.5 hours (IQR: 5.8-9.2) to 3.9 hours (2.8-5.1) (U=1765; p<0.001). Alternative diagnoses decreased from 11.4% to 7.0% (χ²=1.33; p=0.252; RR: 0.62, 95% CI: 0.27-1.43), also a downward trend. These outcome differences between Cycle 1 and Cycle 2 are summarized in Table 1.

**Table 1:** Key Performance Metrics Between Cycle 1 and Cycle 2 Notes: Effect estimates are reported as odds ratios (OR) for RCEM four-hour compliance and relative risk (RR) for other binary outcomes. Continuous variables are presented as median (interquartile range, IQR). p-values correspond to Mann–Whitney U tests for continuous variables and Pearson’s chi-square (χ²) tests for categorical variables, with Fisher’s exact test used when expected cell counts were <5. Two-sided α=0.05. Abbreviations: BAUS, British Association of Urological Surgeons; CT-KUB, computed tomography of kidneys, ureters, and bladder; ED, Emergency Department; IQR, interquartile range; NICE, National Institute for Health and Care Excellence; OR, odds ratio; RCEM, Royal College of Emergency Medicine; RCR, Royal College of Radiologists; RR, relative risk; STONE, Sex, Timing, Origin, Nausea, Erythrocytes

Indicator	Cycle 1	Cycle 2	Absolute Difference	Effect Estimate (95% CI)	p-value (test)	
Urine dip turnaround (min)	144 (98–192)	68 (45–92)	−76	—	<0.001 (U=2102)	
CT request time (min)	166 (120–210)	75 (50–100)	−91	—	<0.001 (U=1850)	
CT report time (min)	124 (90–160)	70 (45–95)	−54	—	<0.001 (U=1924)	
ED stay (hours)	7.5 (5.8–9.2)	3.9 (2.8–5.1)	−3.6	—	<0.001 (U=1765)	
RCEM four-hour compliance (%)	16.7	57	40.3	OR 6.63 (3.58–12.29)	<0.001 (χ²=39.9)	
Serum calcium tested (%)	50.9	65.8	14.9	RR 1.29 (1.03–1.62)	0.022 (χ²=5.27)	
Serum urate tested (%)	0	25.4	25.4	—	<0.001 (Fisher’s)	
STONE score used (%)	5.3	78.9	73.6	RR 15.0 (6.8–32.9)	<0.001 (χ²=127.0)	
Repeat CT < 3 months (%)	19.3	9.6	−9.7	RR 0.50 (0.25–0.98)	0.038 (χ²=4.31)	
Diagnostic yield (stones, %)	57.9	68.4	10.5	RR 1.18 (0.97–1.44)	0.090 (χ²=2.87)	
Alternative diagnoses (%)	11.4	7	−4.4	RR 0.62 (0.27–1.43)	0.252 (χ²=1.33)	

## Discussion

Findings in the clinical context

This two-cycle QIP achieved substantial improvements in timeliness metrics (urine dip, CT request, CT report) and ED throughput, with threefold gains in RCEM four-hour compliance. Biochemical testing rates improved and repeated CT within three months declined. Diagnostic yield trended upward (p=0.09), likely attributable to more selective imaging informed by the STONE score [5,7,9,10,15]. Alternative diagnoses showed a downward trend, indicating that the optimized imaging approach did not impair the identification of non-stone pathologies [5].

Comparison with previous quality initiatives

Our results mirror prior QIPs demonstrating that structured pathways, electronic prompts, and focused education improve imaging timeliness, reduce repeat CTs, and raise diagnostic yield [9-11,13-15]. Initiatives integrating decision support into ordering systems have similarly increased metabolic testing compliance and reduced unwarranted variation [11,12]. Efforts to prioritize transport and hot reporting in radiology align with observed reductions in total ED length of stay [13,14]. For instance, a similar two-cycle QIP increased CT-KUB utilization from 56% to 73%, with comparable reductions in repeat imaging [11]. Another audit improved serum calcium and urate testing rates to over 60% through education and prompts, mirroring our gains but achieving higher urate testing (up to 80%) via standardized blood panels [12]. ED length-of-stay reductions (from 7.5 to 3.9 hours) align with initiatives introducing dedicated colic clinics or pathways, which reported decreases of two to four hours and improved referral times.

Strengths

Strengths include consecutive inclusion with consistent eligibility, multidisciplinary design embedded in routine workflows, frequent governance feedback with fidelity checks, and complete, consistent statistical reporting (test statistics, effect estimates, and CIs).

Limitations 

Limitations include a single-center, retrospective pre-post design without concurrent controls; possible seasonal effects; lack of formal normality assessment (mitigated by nonparametric testing); and omission of analgesia timing metrics (e.g., NSAID administration per NICE QS195 [3]). While a priori targets promote transparency and operational focus, they may not fully reflect baseline process capability; therefore, effect sizes, confidence intervals, and p-values, rather than target attainment, underpin the interpretation of outcomes.

Implications and future directions

Low-cost, scalable measures - STONE prompts, registrar-initiated CT, porter prioritization, and hot reporting - can deliver rapid performance gains. Adopting a standardized “stone profile” (serum calcium, urate) and exploring low-dose CT protocols may enhance safety and adherence. Future work should examine sustainability, patient-centered outcomes (pain control, revisits), and generalizability across multiple sites.

## Conclusions

This two-cycle QIP demonstrated that targeted, low-cost interventions can achieve substantial improvements in the emergency management of acute renal colic. Implementation of a “Straight-to-CT” pathway, mandatory STONE score documentation, and workflow optimization significantly enhanced compliance with NICE, BAUS, RCR, and RCEM standards, reduced diagnostic delays, shortened ED length of stay, and improved rates of metabolic testing. These gains were realized without compromising diagnostic yield for alternative pathology and were associated with fewer repeat CT scans, thereby reducing unnecessary radiation exposure.

The clinical implications are clear. Embedding pragmatic workflow adjustments - such as ED registrar-initiated “Straight-to-CT” pathways, integrated electronic STONE score prompts, and hot reporting - can markedly improve ED performance within existing resources. These strategies are scalable within existing resources and align with national standards; however, results should be interpreted as associations, pending multicentre or controlled evaluations. Sustained monitoring, adoption of a standardized biochemical “stone profile,” and implementation of low-dose CT protocols may further enhance patient outcomes while promoting responsible resource utilization. Future work should focus on multi-center adoption to confirm generalizability, incorporate patient-centered outcomes such as pain control and re-attendance, and evaluate long-term sustainability. By integrating evidence-based guidelines with pragmatic workflow redesign, emergency departments can provide faster, safer, and more consistent care for patients presenting with suspected renal colic.
